# Dr. Lombe Atthill

**Published:** 1910-09-24

**Authors:** 


Dr. Lombe Atthill, of Monkstown Castle, Co. Dublin, died on the platform of Strood Railway Station, Rochester, last week. He was eighty-four, and qualified as L.R.C.S.I. and L.M. in 1847, later taking the degrees of M.B. and M.D. at Dublin University, becoming also F.R.C.P.I. He had been President of the Royal Academy of Medicine of Ireland, the Royal College of Physicians of Ireland, and the Dublin Obstetrical Society. He had also been Master of the Rotunda Hospital, Dublin, and was an honorary Fellow of the Edinburgh Obstetrical Society and an honorary member of the Gynæcological Society of Boston. His best known work is his book of "Lectures on Diseases Peculiar to Women."

